# Supplementary material for: A Microfluidic Approach for Probing Heterogeneity in Cytotoxic T-Cells by Cell Pairing in Hydrogel Droplets
Source: Micromachines (Basel). 2022 Nov 4;13(11):1910. doi: 10.3390/mi13111910 (PMC9692327; doi:10.3390/mi13111910)
Supplement: Supplementary file 1 [file micromachines-13-01910-s001.zip › supplementary figures.pdf]

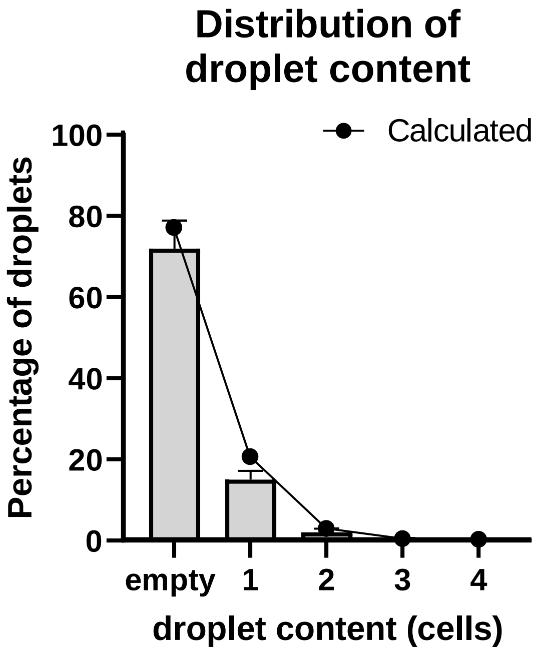

Supplementary figure S1: Droplet characterization. Quantified droplet contents as compared to Poisson distributed calculated expected values. 1463 droplets were counted from 4 independent batches of droplets.

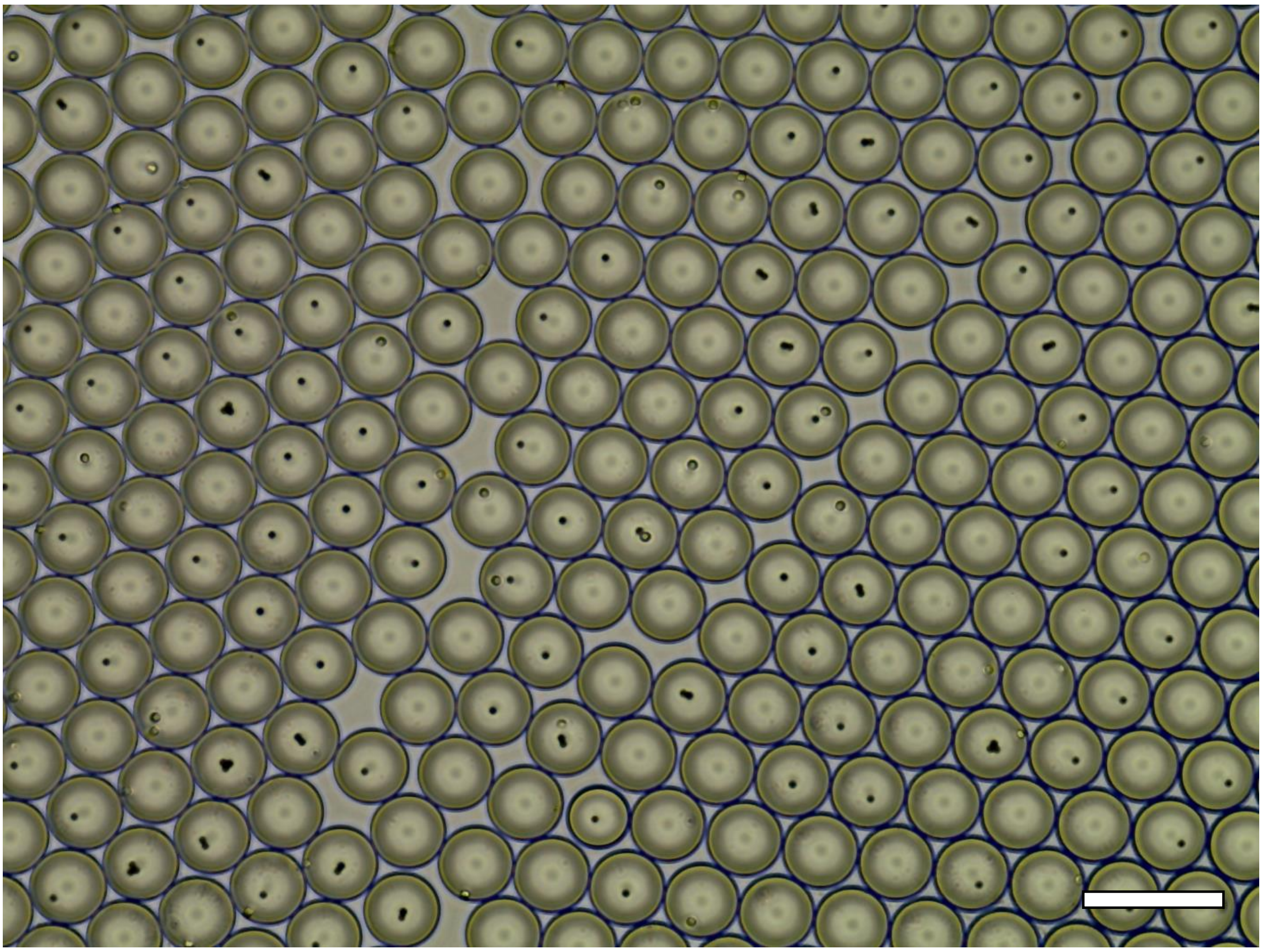

Supplementary figure S2: CTL and aAPC distribution over monodisperse aqueous droplets. Scale bar represents 100µm.

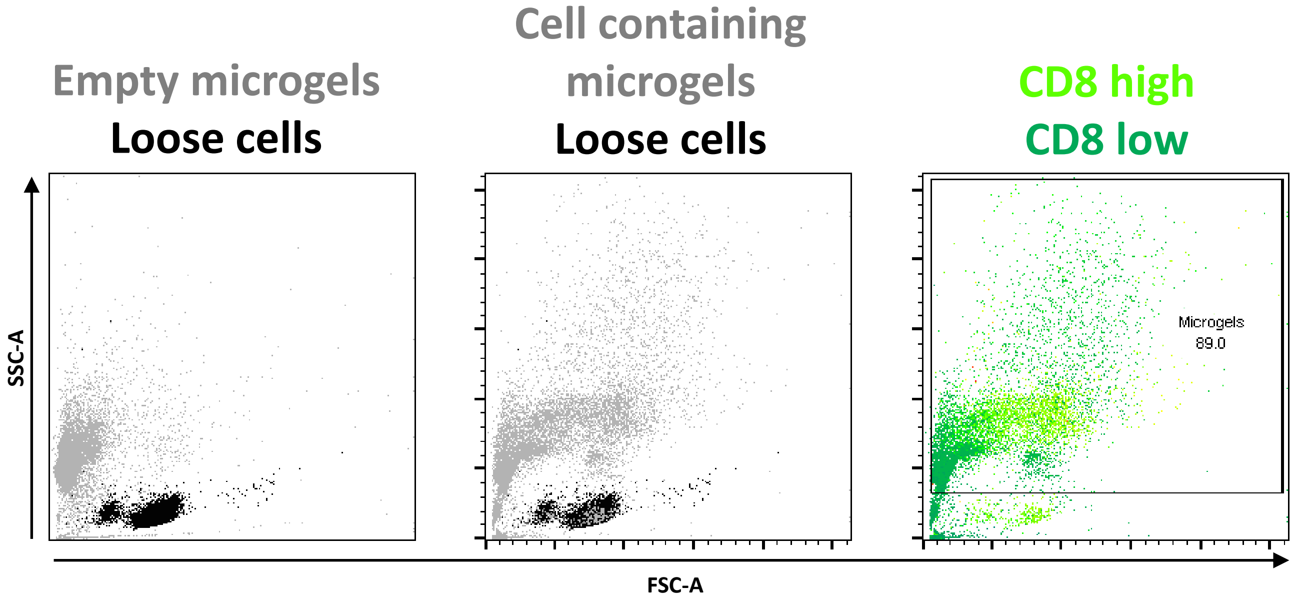

Supplementary figure S3:Flow cytometry analysis of microgels, FSC-A vs SSC-A. Left to right: microgels without cells encapsulated vs. cells without microgel, microgels with cells encapsulated vs. cells without microgel, heatmap colored plot of microgels with cell encapsulated.

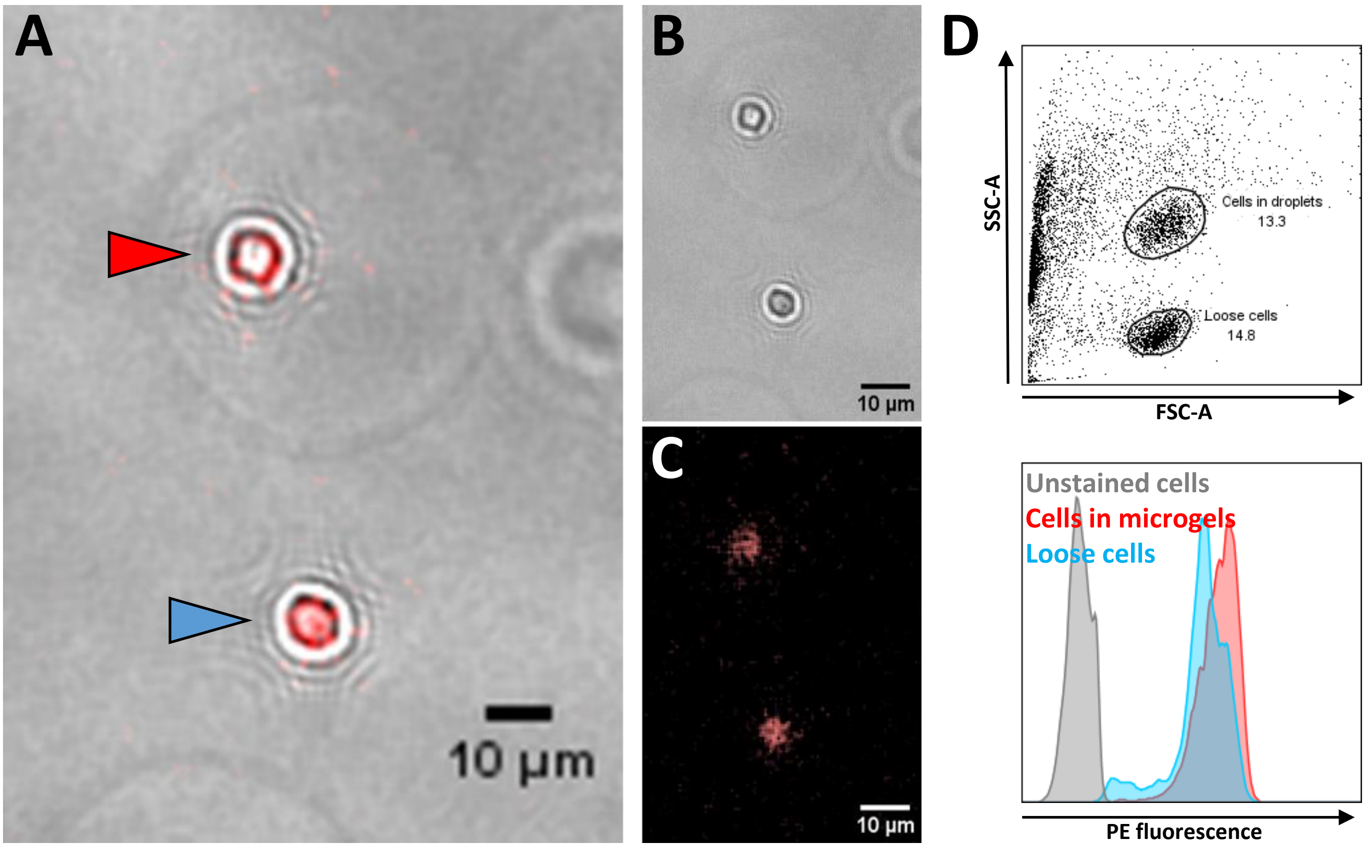

Supplementary figure S4: Fluorescent staining of cells inside and outside of microgels stained for PE fluorescence. A) overlay microscopy image of cell in microgels (red arrow) and cell outside of microgel (blue arrow) and B) brightfield and C) PE channel separate. D) Flow cytometric analysis of the same sample showing cells are stained with indistinguishable fluorescent intensity inside or outside of microgels.

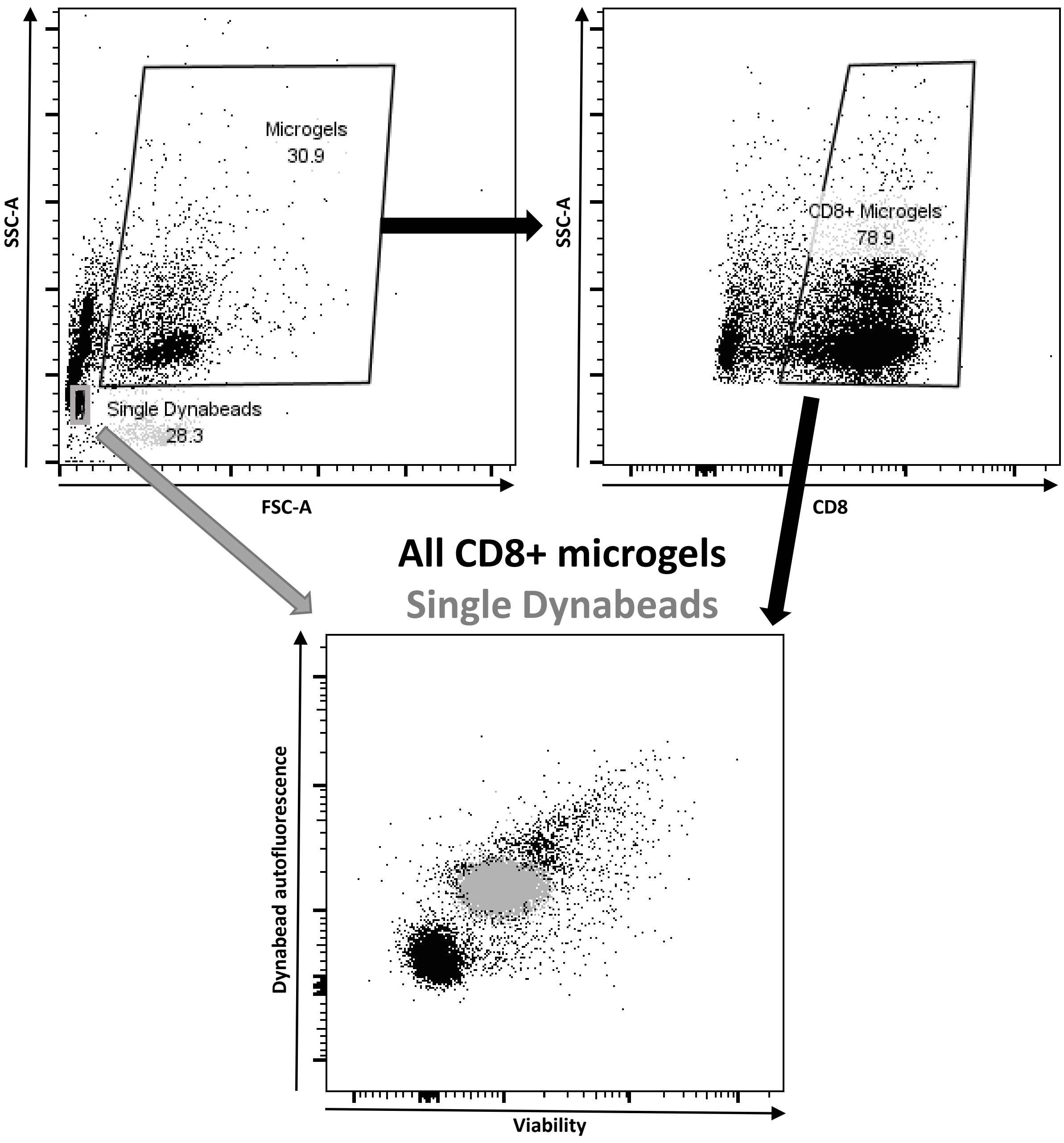

Supplementary figure S5: Choosing gating strategy for 1:1 pair selection. From total events single Dynabeads are selected and microgels containing CD8+ cells. In a dot plot overlay the population of CD8+ microgels containing exactly one Dynabead can be easily recognized.

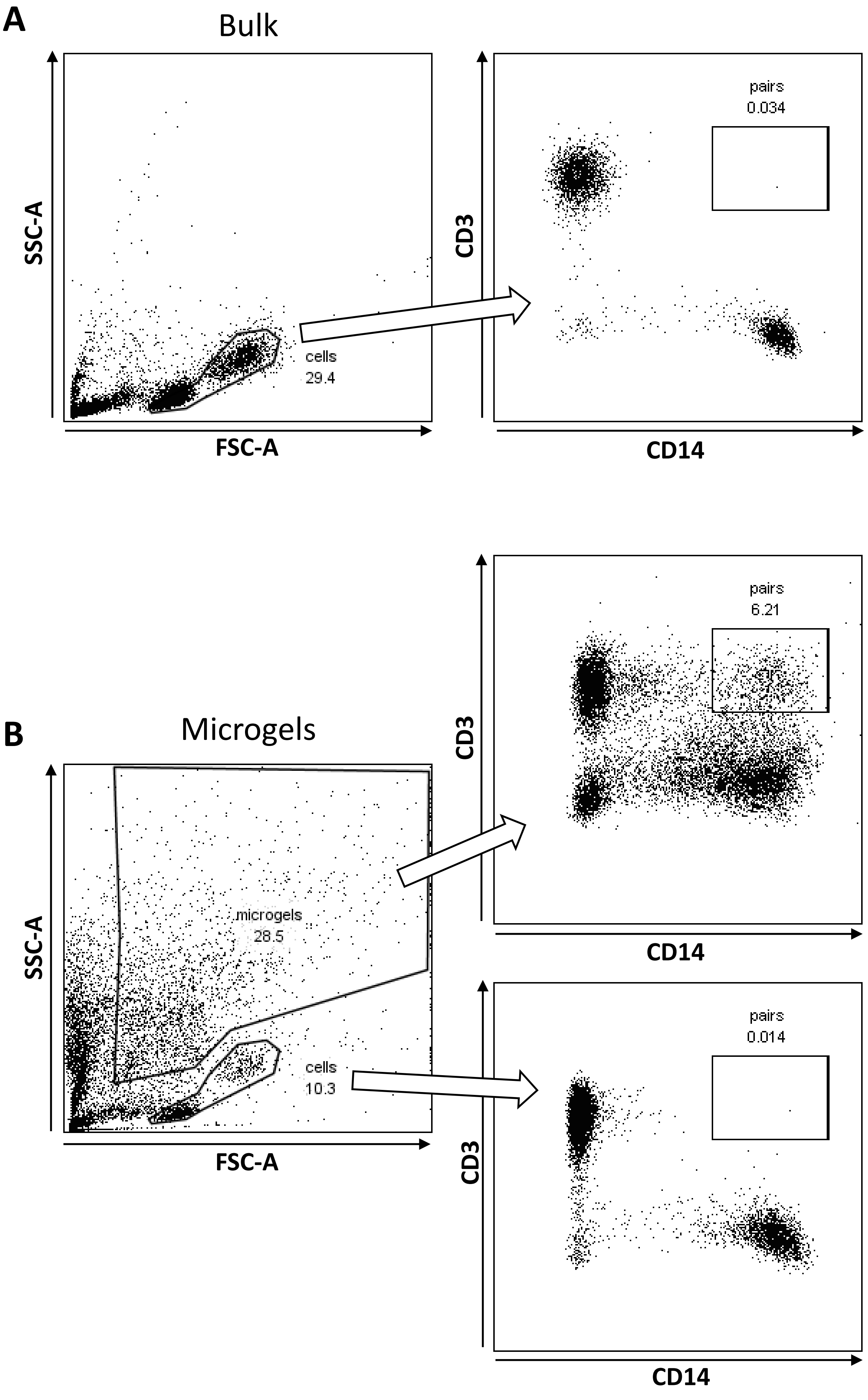

Supplementary figure S6: Monocyte and T-cell pairing. A) bulk cultured mix of T-cells and Monocytes and their CD3 and CD14 expression, no double positive events can be observed. B) Mix of T-cells and monocytes both in microgels and bulk and their CD3 and CD14 expression. Double positive events can only be observed for cells in microgels.
